# Minimally-invasive debulking of ovarian cancer in the rat pelvis by means of photodynamic therapy using the pegylated photosensitizer PEG-m-THPC

**DOI:** 10.1038/sj.bjc.6690740

**Published:** 1999-10

**Authors:** R Hornung, M K Fehr, J Monti-Frayne, B J Tromberg, M W Berns, Y Tadir

**Affiliations:** Beckman Laser Institute and Medical Clinic, University of California Irvine, 1002 Health Sciences Road E, Irvine, CA, 92612, USA; Department of Obstetrics and Gynaecology, University Hospital Zurich, Switzerland

**Keywords:** interstitial photodynamic therapy, ovarian cancer, PEG-m-THPC, minimally invasive, rat

## Abstract

Interstitial photodynamic therapy (PDT) using the pegylated photosensitizer PEG-m-THPC was evaluated as a minimally-invasive procedure to selectively debulk unrespectable pelvic ovarian cancer (NuTu-19) in immunocompetent rats. To assess tumour selectivity, PEG-m-THPC at dosages of 0.3, 3.0 and 30 mg kg^−1^ body weight was administered intravenously to 30 rats 4 weeks following tumour induction. Eight days later laser light at 652 nm and optical doses ranging from 100 to 900 J cm^−1^ diffuser-length was delivered by an interstitial cylindrical diffusing fibre inserted blindly into the pelvis. Three days following light application, the volume of necrosis was measured and the damage to pelvic organs was assessed histologically on cross sections. For analysis of survival, 20 tumour-bearing rats received PDT using drug doses of 3 or 9 mg kg^−1^ body weight and an optical dose of 900 J cm^−1^ diffuser-length, whereas ten untreated tumour-bearing rats served as controls. The histological assessment of PDT induced necrosis showed a non-linear dose–response for both the photosensitizer dose and the optical dose. The lowest drug dose activated with the highest optical dose did not induce more necrosis than seen in tumour-bearing control animals. The same optical dose induced necrosis of 17 mm in diameter using 30 mg kg^−1^ and 11 mm using 3 mg kg^−1^ photosensitizer. The optical threshold for induction of significant necrosis was between 100 and 300 J cm^−1^ diffuser-length for 30 mg kg^−1^ and between 300 and 500 J cm^−1^ for 3 mg kg^−1^ PEG-m-THPC. Significant damage to normal pelvic organs was only seen if 30 mg kg^−1^ photosensitizer was activated with optical doses of 700 J cm^−1^ or more. In the survival study, all treated animals survived PDT for at least 2 weeks and the intestinal and urinary tract remained functional. No clinical signs of blood vessel or nerve injury were observed. Mean overall survival of untreated tumour-bearing rats was 25.0 ± 4.5 days compared to 38.4 ± 3.8 days and 40.0 ± 3.6 days for rats treated with 3 mg kg^−1^ or 9 mg kg^−1^ PEG-m-THPC mediated PDT respectively (*P* < 0.05). We conclude that PEG-m-THPC mediated PDT has a favourable therapeutic window and that this minimally-invasive procedure can reduce pelvic cancer bulks effectively and selectively. © 1999 Cancer Research Campaign


*British Journal of Cancer* (1999) **81**(4), 631–637
© 1999 Cancer Research Campaign
Article no. bjoc.1999.0740

### Minimally-invasive debulking of ovarian cancer in the rat pelvis by means of photodynamic therapy using the pegylated photosensitizer PEG-m-THPC

**R Hornung[1,2], MK Fehr[2], J Monti-Frayne[1], BJ Tromberg[1], MW Berns[1] and Y Tadir[1]**

[1]Beckman Laser Institute and Medical Clinic, University of California Irvine, 1002 Health Sciences Road E, Irvine, CA 92612, USA; [2]Department of Obstetrics and Gynaecology, University Hospital Zurich, Switzerland

**Summary** Interstitial photodynamic therapy (PDT) using the pegylated photosensitizer PEG-m-THPC was evaluated as a minimally-invasive procedure to selectively debulk unrespectable pelvic ovarian cancer (NuTu-19) in immunocompetent rats. To assess tumour selectivity, PEG-m-THPC at dosages of 0.3, 3.0 and 30 mg $kg^{-1}$ body weight was administered intravenously to 30 rats 4 weeks following tumour induction. Eight days later laser light at 652 nm and optical doses ranging from 100 to 900 J $cm^{-1}$ diffuser-length was delivered by an interstitial cylindrical diffusing fibre inserted blindly into the pelvis. Three days following light application, the volume of necrosis was measured and the damage to pelvic organs was assessed histologically on cross sections. For analysis of survival, 20 tumour-bearing rats received PDT using drug doses of 3 or 9 mg $kg^{-1}$ body weight and an optical dose of 900 J $cm^{-1}$ diffuser-length, whereas ten untreated tumour-bearing rats served as controls. The histological assessment of PDT induced necrosis showed a non-linear dose–response for both the photosensitizer dose and the optical dose. The lowest drug dose activated with the highest optical dose did not induce more necrosis than seen in tumour-bearing control animals. The same optical dose induced necrosis of 17 mm in diameter using 30 mg $kg^{-1}$ and 11 mm using 3 mg $kg^{-1}$ photosensitizer. The optical threshold for induction of significant necrosis was between 100 and 300 J $cm^{-1}$ diffuser-length for 30 mg $kg^{-1}$ and between 300 and 500 J $cm^{-1}$ for 3 mg $kg^{-1}$ PEG-m-THPC. Significant damage to normal pelvic organs was only seen if 30 mg $kg^{-1}$ photosensitizer was activated with optical doses of 700 J $cm^{-1}$ or more. In the survival study, all treated animals survived PDT for at least 2 weeks and the intestinal and urinary tract remained functional. No clinical signs of blood vessel or nerve injury were observed. Mean overall survival of untreated tumour-bearing rats was 25.0 ± 4.5 days compared to 38.4 ± 3.8 days and 40.0 ± 3.6 days for rats treated with 3 mg $kg^{-1}$ or 9 mg $kg^{-1}$ PEG-m-THPC mediated PDT respectively ($P < 0.05$). We conclude that PEG-m-THPC mediated PDT has a favourable therapeutic window and that this minimally-invasive procedure can reduce pelvic cancer bulks effectively and selectively. © 1999 Cancer Research Campaign

**Keywords:** interstitial photodynamic therapy; ovarian cancer; PEG-m-THPC; minimally invasive; rat

Malignant pelvic tumours (including carcinoma of the colon, the prostate, the bladder, the cervix, the uterine endometrium and the ovaries) contribute to 36% of all new cancer cases and 16% of all cancer related deaths (Parker et al, 1996). Ovarian cancer represents 5% of cancer deaths in women but is the cause of the greatest number of gynaecological deaths in the developed world (Boring et al, 1994). The lack of symptoms in the early stages of ovarian cancer means that up to 80% of newly diagnosed patients will have disease that is advanced and often not totally resectable. The prognosis of these women is poor, with about 20% surviving for 5 years after diagnosis (Pettersson, 1995).

A mainstay of treatment is surgical tumour debulking since survival is significantly improved if optimal surgical cytoreduction is achieved prior to chemotherapy (Curtin et al, 1997; Munkarah et al, 1997). Unfortunately, two-thirds of the patients with advanced ovarian cancer cannot be optimally debulked (Schwartz, 1997) because of unresectable, bulky tumours in the cul-de-sac or upper abdomen, or due to retroperitoneal tumours adherent to major abdominal vessels (Gershenson, 1994). Electrocautery (Deppe et al, 1986), argon beam coagulator (Brand and Pearlman, 1990), Cavitron Ultrasonic Surgical Aspirator (CUSA) (Deppe et al, 1990) and neodymium-yttrium-aluminium-garnet (Nd-YAG) lasers (Brand et al, 1988) are used to improve cytoreductive surgery. Most of these techniques require an open surgical procedure, none are selective for cancerous tissue, and their beneficial impact on cytoreduction remains unproven (Gershenson, 1994). Hence, a new tumour debulking technique, suitable for open surgical procedures in curative intent, or for minimally-invasive palliative procedures is of the utmost clinical importance.

The importance of photodynamic therapy (PDT) in the treatment of malignant tumours is currently being evaluated. PDT is based on the preferential uptake and/or retention of a photosensitizer by malignant tissues (Gomer and Dougherty, 1979; Barr et al, 1990; Chatlani et al, 1992). Irradiation of the tissue containing the photosensitizer with light of appropriate wavelength and energy leads to oxidation-mediated tissue necrosis (Weishaupt et al, 1976; Kimel et al, 1989). Selective tumour destruction can be achieved in three ways: (1) administering a drug that preferentially localizes in tumour tissue; (2) applying a drug that distributes similarly in all tissues but PDT affects the tumour more than normal tissues; and (3) focusing the light on the tumour only (Moan and Berg, 1992). In ovarian cancer promising attempts to use PDT for

*Received 15 January 1999*
*Revised 22 March 1999*
*Accepted 31 March 1999*

*Correspondence to:* Y Tadir disseminated small peritoneal metastasis have been proposed (Tochner et al, 1985, 1986, 1991; Delaney and Glatstein, 1988; DeLaney et al, 1991, 1993; Sindelar et al, 1991; Goff et al, 1994, 1996; Veenhuizen et al, 1994; Molpus et al, 1996; Lilge et al, 1998). However, the geometric complexity and the large surface of the human peritoneum make light dosimetry for intraperitoneal PDT difficult. Furthermore, chemotherapy is an effective treatment modality for diffuse, small residual cancer nodules persisting after surgical debulking. In contrast, the potential of PDT to debulk non-resectable pelvic cancer in a minimally-invasive procedure has not yet been evaluated.

Many photosensitizers have been evaluated for their ability to preferentially localize in malignant tissue, commonly expressed as the tumour to tissue ratio (TTR). Porphyrin derivatives are the most commonly administered photosensitizers. Modifications to the porphyrin structure (Bonnett and Berenbaum, 1989; Bonnett et al, 1989) have produced the so-called second-generation photosensitizers, including the phthalocyanines (Ben-Hur and Rosenthal, 1985; Rosenthal, 1991) and chlorins (Gomer, 1991) with TTR values ranging from 1:1 to 5:1 (Pass, 1993). Meso-tetra(hydroxyphenyl)chlorin (m-THPC) is a chlorin photosensitizer with promising properties. M-THPC has been found to be extremely effective in animal tumour models, as well as in clinical trials (Bonnett and Berenbaum, 1989; Ris et al, 1991; 1993*a*, 1993*b*; Peng et al, 1995; Wierrani et al, 1997) and it shows selective uptake by malignant tissues. However, preparation of this photosensitizer in aqueous solution for systemic application is difficult due to its hydrophobicity. The addition of four long hydroxyl (polyethylene glycol, PEG 2000) side-chains linked to m-THPC through a triazine group produces a tetrakis-(m-methoxypolyethylene glycol) derivative of 7,8-dihydro-5,10,15,20-tetrakis(3-hydroxyphenyl)-21-23-[H]-porphyrin (PEG-m-THPC). PEG-m-THPC is characterized by high hydrophilicity and lack of instability in vitro (Grahn et al, 1997). The molecular weight of this water soluble compound is 6515 Daltons compared to 680 Daltons for its hydrophobic parent sensitizer, m-THPC. The relatively large size and the hydrophilicity of this pegylated compound make it a unique photosensitizer. In a fluorescence microscopy study (manuscript in preparation) we demonstrated highly selective targeting of cancer tissue by PEG-m-THPC in an immunocompetent rat ovarian cancer model. Tumour fluorescence was maximal 8 days following intravenous injection of PEG-m-THPC and approximately 40 times higher than fluorescence of most abdominal organs.

The aim of this study is twofold: first to assess in a histological study the selectivity of debulking pelvic ovarian cancer masses in the rat by PEG-m-THPC mediated interstitial PDT. Second to evaluate the impact of this minimally-invasive treatment on the survival of rats bearing bulky pelvic ovarian cancers.

### MATERIALS AND METHODS

### Tumour model

Seventy-two pathogen-free female Fischer 344 rats (Harlan Sprague Dawley, Inc., IN, USA), weighing 220 g (± 24 s.d.) were housed in a pathogen-free animal facility and given commercial basal diet and water ad libitum. The study was approved by the Institutional Laboratory Animal Care and Use Committee, University of California, Irvine.

The NuTu-19 cell line is a poorly-differentiated epithelial ovarian cancer cell line derived from the Fischer 344 rats (Testa et al, 1994). This syngeneic rat tumour model was chosen because these cells grow and metastasize in immunocompetent rats in the same way epithelial ovarian cancers do in humans (Rose et al, 1996).

Cells were cultured in RPMI-1640 medium (Gibco Life Technologies, Grand Island, NY, USA) enriched with 10% fetal calf serum (FCS; Gemini Bioproducts, Calabassas, CA, USA), 25 IE $ml^{-1}$ penicillin and 25 mg $ml^{-1}$ streptomycin and incubated under standardized conditions (37°C, 7% carbon dioxide, 100% humidity). The NuTu-19 cells were harvested with 0.25% trypsin (Gibco Life Technologies) from tissue culture flasks and washed with phosphate-buttered saline (PBS; Gibco Life Technologies). A total of 20 million cells was injected into the caudal part of the right psoas muscle through a 15 mm lower median laparotomy which was closed in two layers. Four weeks following tumour inoculation bulky pelvic tumours measuring 2.5 cm in diameter developed. Tumour masses protruded out of the osseous pelvis leading to tumour volumes greater than that of the normal rat pelvis. All invasive procedures were carried out under isoflurane (Forane, Ohmeda PPD Inc. Liberty Corner, NJ, USA) -oxygen gas anaesthesia. Five millilitres lactated Ringer solution (Abott Laboratories, North Chicago, IL, USA) was given subcutaneously (s.c.) prior to laparotomy or PDT and 0.5 ml enrofloxacin (Baytril, Bayer Corp., Shawnee Mission, KA, USA) was injected s.c. prior to surgery or following PDT. During all procedures, body temperature was kept constant using a heating pad.

### PDT

The photosensitizer, PEG-m-THPC, was kindly provided by Scotia Pharmaceuticals Ltd (Guildford, UK; SC 102, Batch # HR/1/063). The crystalline photosensitizer was dissolved in distilled water to concentrations ranging from 25 to 0.25 mg $ml^{-1}$ (corresponding to 2.6–0.026 mg $ml^{-1}$ of equimolar concentrations of active m-THPC), and sterilized by microfiltration through a 0.22-μm filter unit (Millex-GS, Millipore Corp. Bedford, MA, USA).

Four weeks following tumour induction, 0.3, 3, 9, or 30 mg PEG-m-THPC-powder per kg body weight was injected into the tail vein. Eight days following drug application photoactivation was performed as follows. Through a 2 mm skin incision, a 13-gauge needle was blindly inserted paramedian right, parallel to the vertebral column, piercing the anterior abdominal wall, through the tumour and the entire pelvis until the needle tip arrived underneath the skin close to the tail. A cylindrical light diffuser (25-mm length, 1.6-mm diameter coupled to a 0.7-mm optical fibre; Optiguide Fibre Optic DCYL25, QLT PhotoTherapeutics Inc., Seattle, WA, USA) was placed in the pelvis through the needle. The needle was then withdrawn, placing the cylindrical light diffuser in direct contact with the tissues. Laser-light, generated by an argon-pumped dye laser (Spectra-Physics 171 and Spectra-Physics Model 375, Mountain View, CA, USA) was launched into the diffusing fibre. The laser was tuned to 652 nm as verified by a clinical Hartridge Reversion Spectroscope (Ealing Electro-Optics, South Natick, MA, USA). The power was measured using an integrating sphere (Intragold IS 060, LabSphere, North Houston, NH, USA) coupled to a power meter (model 210, Coherent Corp., Palo Alto, CA, USA) and kept constant at 150 mW $cm^{-1}$ diffuser-length which is below threshold levels for thermal damage (Lowdell et al, 1993).

© 1999 Cancer Research Campaign

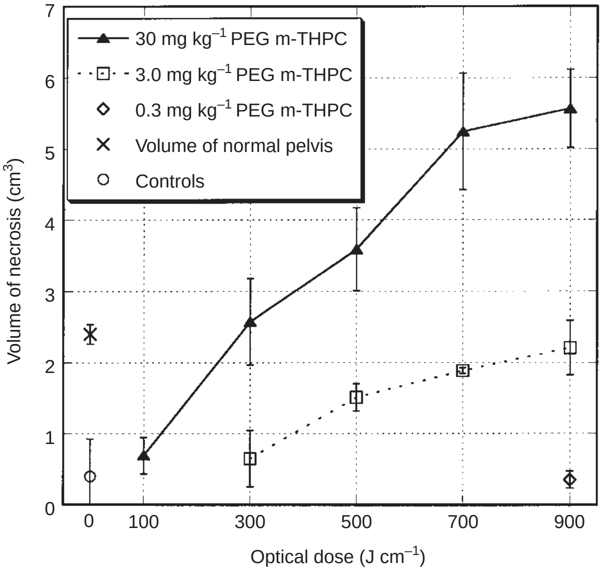

**Figure 1** The mean volume (cm$^3$) of necrosis ± s.e.m. in the rat pelvis is displayed as a function of the optical dose of laser light (J cm$^{-1}$). Data are shown separately for three different PEG-m-THPC concentrations (30 mg kg$^{-1}$, 3 mg kg$^{-1}$, 0.3 mg kg$^{-1}$). Spontaneous necrosis of tissues in tumour-bearing rats which were not exposed to either PEG-m-THPC or laser light are shown as a circle at the optical dose '0 J cm$^{-1}$' (controls). 'X' represents the mean volume of the normal rat pelvis (at 0 J cm$^{-1}$). $n$ = 3 rats per given optical dose (exception: at 900 J cm$^{-1}$ and 30 mg kg$^{-1}$ $n$ = 2)

The light dose for interstitial PDT is usually quoted as J cm$^{-1}$ (Lowdell et al, 1993). Pelvic tumours were exposed to various optical doses of laser light (100, 300, 500, 700 or 900 J cm$^{-1}$ diffuser-length) by varying exposure time from 11 to 100 min.

### Histological study

Thirty-nine rats were used for histological assessment of necrosis. Following drug doses were used: 30, 3 and 0.3 mg kg$^{-1}$ PEG-m-THPC. The photosensitizer was activated with either 100, 300, 500, 700 or 900 J cm$^{-1}$ laser-light in rats sensitized with 30 mg kg$^{-1}$ PEG-m-THPC. Optical doses of either 300, 500, 700 or 900 J cm$^{-1}$ were used in rats sensitized with 3.0 mg kg$^{-1}$ PEG-m-THPC, and an optical dose of 900 J cm$^{-1}$ was used to activate 0.3 mg kg$^{-1}$ PEG-m-THPC. At least three rats were used for every drug–optical dose combination. Four rats were treated with an optical dose of 900 J cm$^{-1}$ and 30 mg kg$^{-1}$ photosensitizer. Two out of these four rats died during or immediately after PDT and were not used for histology. Three tumour-bearing rats were used as controls and not exposed to PDT.

Three days following PDT, the rats were sacrificed with an intracardiac injection of Eutha-6 (Western Medical Supply, Arcadia, CA, USA). In order to study PDT effects on both tumour and normal pelvic organs the entire lower abdomen, including the tumour, all pelvic organs, and the abdominal wall without skin was removed 'en-bloc'. Following fixation and decalcification (Rapid Bone Decalcifier RDO, Apex Engineering Products Corporation, Plaintiel, IL, USA) five transversal, 6- to 8-mm thick sections were taken from each pelvis, sectioned to 6-µm thick slices, and stained with haematoxylin and eosin (H&E).

Histology slides were analysed for both the area of PDT-induced necrosis and for damage to normal tissues. The overall damage was defined as the volume of necrosis including adjacent perinecrotic inflammation. The margins of necrosis were marked with a permanent marker. Histology slides and a ruler were scanned (Umax Vista-SGE, Umax Data Systems Inc., Hsinchu, Taiwan; Photoshop, Adobe Systems Inc, Mountain View, CA, USA) into a computer. The area of PDT induced necrosis was measured using microscope-calibrated image processing software (IPlab software, Signal Analytic, Vienna, VA, USA). The average area of necrosis was calculated from at least three transversal sections per rat. The volume (cm$^3$) of necrosis was calculated from the product of the mean necrotic area (cm$^2$) and the length of the cylindrical light diffuser (cm). To compare the volume of PDT-induced necrosis to the volume of a normal rat pelvis, the average volume of the osseous pelvic cavity was calculated in four non-tumour-bearing rats.

In order to assess selectivity of PDT, damage to normal organs was determined by a numerical scoring system. Each of the pelvic organs (i.e. colon, ureters, urinary bladder, vagina, uterus, major abdominal and pelvic vessels, pelvic muscles and interstitial connective tissues) were scored as follows: 0 = no damage; 1 = severe oedema; 2 = necrosis of the muscle *or* mucosal layer; 3 = necrosis of the muscle *and* mucosal layer. Each organ was scored on five transversal sections. In paired organs the highest score attributed to a specific organ was multiplied by a factor of two, if bilateral damage was found, assuming the worst case. In unpaired organs the highest score attributed was multiplied by a factor of two, if more than 50% of the organ circumference was damaged in order to quantify the extent of damage. In addition, scores were multiplied by a factor of two, if damage to a specific organ was seen in more than one of the five transversal planes through the pelvis. The sum of all organ scores per rat was calculated and divided by the greatest possible damage score to express the relative damage per rat. Relative damage was expressed with a 0–1 scoring range with 1 indicating all organs were destroyed, and e.g. 0.3 indicating 30% of the greatest possible damage was found.

### Survival study

In order to assess the impact of PDT on the functional integrity of the pelvic organs and on tumour progression, 30 rats were randomly assigned to either 'control' (tumour-bearing but no PDT, $n$ = 10), or 'treatment' groups (3 or 9 mg kg$^{-1}$ PEG-m-THPC; optical dose 900 J cm$^{-1}$ each; $n$ = 10 per group). Animals were kept alive without special protection from room light, and checked for weight, eye colour, defecation, urination and behaviour daily (data not shown). The study end point was defined as spontaneous death or sacrifice due to following ethical reasons: increase or decrease of body weight by more than 10% over a period of 3 days in combination with either anaemic eyes or lack of defecation or urination for at least 3 days. Based on these clinical symptoms, severe cancer-related complications such as haemorrhagic ascites and/or obstruction of the intestinal or urinary pathways or rejection of food were assumed and sacrifice was indicated.

### Data analysis

The volume of necrosis and the score of relative damage to normal organs were averaged for three rats per given photosensitizer and optical dose. Data are presented as mean values ± standard error. For statistical analysis of differences in tumour necrosis at various

© 1999 Cancer Research Campaign optical doses or damage scores at various optical doses the Kruskal–Wallis test was used. If a significant overall difference was present, multiple comparisons were performed using the Bonferroni–Dunn multiple comparison procedure. Cumulative survival was calculated using the Kaplan–Meier survival analysis (Kaplan and Meier, 1958). Statistical significance for survival analysis was calculated using the Peto-Peto–Wilcoxon test. *P*-values < 0.05 were considered significant for all statistics.

### RESULTS

Volumes of PDT-induced necrosis are shown in Figure 1 as a function of optical dose (J cm$^{-1}$) for the three drug concentrations (30, 3 and 0.3 mg kg$^{-1}$ PEG-m-THPC) used in the histological study. Spontaneous necrosis in tumour bearing rats exposed to neither drug nor light measured 0.4 ± 0.5 cm$^3$ and is referred to as the optical dose '0 J cm$^{-1}$'. The mean volume of four normal rat pelvises was 2.4 ± 0.14 cm$^3$ and is shown at the position '0 J cm$^{-1}$'. The lowest drug dose (0.3 mg kg$^{-1}$ PEG-m-THPC) activated with the highest optical dose (900 J cm$^{-1}$) did not induce more necrosis than found in tumour-bearing control animals. We conclude that 0.3 mg kg$^{-1}$ PEG-m-THPC does not induce photosensitization and 900 J cm$^{-1}$ laser light does not induce relevant thermal damage. A tenfold higher photosensitizer concentration (3 mg kg$^{-1}$ PEG-m-THPC) activated with the same optical dose (900 J cm$^{-1}$) induced an overall damage close to the volume of the treated pelvises. Using the highest drug concentration (30 mg kg$^{-1}$ PEG-m-THPC) and the highest optical dose (900 J cm$^{-1}$), the volume of necrosis doubled that of a normal pelvis. The volume of necrosis can exceed that of the anatomical pelvis since the tumour-bulk protrudes out of the osseous pelvis. Differences between volumes of necrosis induced with the three dosages of PEG-m-THPC were statistically significant ($P < 0.05$) at any given optical dose. For the highest optical dose used, the depth of necrosis measured from the surface of the light diffuser was 8.5 mm with 30 mg kg$^{-1}$, and 5.5 mm with 3 mg kg$^{-1}$. The optical threshold for induction of significant necrosis was between 100 and 300 J cm$^{-1}$ diffuse length for the highest drug concentration, and between 300 and 500 J cm$^{-1}$ for 3 mg kg$^{-1}$ PEG-m-THPC. Increasing the optical dose resulted in a non linear dose–response. An optical dose above 700 J cm$^{-1}$ diffusing-fibre did not substantially increase the volume of necrosis.

The impact of PEG-m-THPC mediated PDT on normal pelvic organs is displayed in Figure 2 as a function of the optical dose for the three drug concentrations used. Spontaneous damage to organs, such as tissue destruction by tumour infiltration, is shown

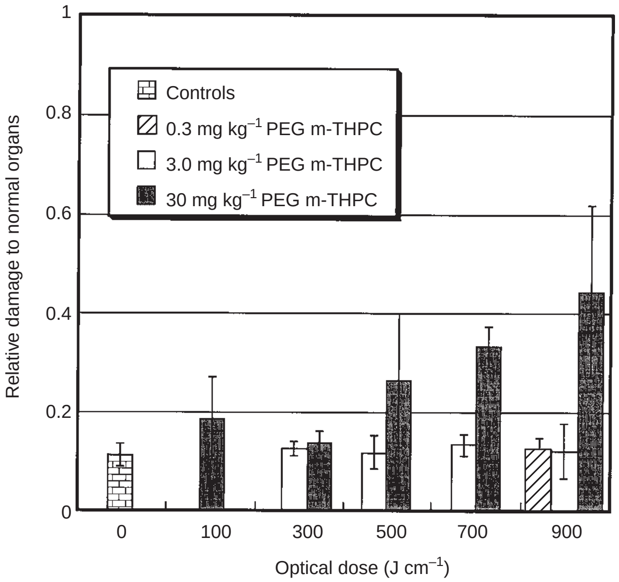

**Figure 2** The impact of PEG-m-THPC sensitized minimally-invasive PDT on normal pelvic organs (i.e. colon, ureters, urinary bladder, vagina, uterus, major abdominal and pelvic blood vessels, pelvic muscles, and interstitial connective tissue) was assessed on cross sections through the pelvises of tumour-bearing rats. The relative damage to normal pelvic organs (score of the examined rat divided by the greatest possible damage score) is shown as a function of the optical dose (J cm$^{-1}$). Scores for various concentrations (30, 3, 0.3 mg kg$^{-1}$) of the photosensitizer PEG-m-THPC are shown. Spontaneous damage to organs, such as tissue destruction by tumour infiltration, are shown at the position '0 J cm$^{-1}$' (controls). $n = 3$ rats per given optical dose (exception: at 900 J cm$^{-1}$ and 30 mg kg$^{-1}$ $n = 2$)

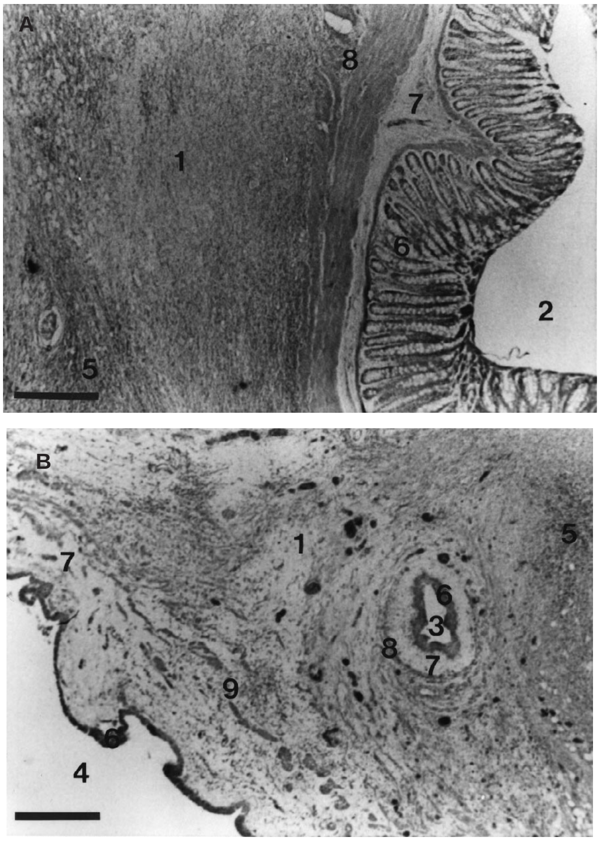

**Figure 3** Two representative H&E-stained micrographs (10× magnification) of cross sections through the lower abdomen of a rat sacrificed 3 days after minimally-invasive PDT of a bulky pelvic tumour are shown. The animal was treated with 700 J cm$^{-1}$ fibre-length of laser-light at 652 nm 8 days following intravenous injection of 30 mg kg$^{-1}$ PEG-m-THPC. Both micrographs show completely necrotic tumour masses (1) encompassing normal pelvic organs such as the colon (2), the ureter (3), and the urinary bladder (4). A strong inflammatory reaction is infiltrating the necrosis (5). Although the light-diffuser was in immediate vicinity of the pelvic organs, they show intact epithelial layers (6) with moderate oedema of the underlying connective tissues (7). The muscle layers (8) of the colon and the ureter are histologically intact and neither megacolon nor hydroureter can be seen, suggesting that the smooth muscles remained functional. The muscle layers of the urinary bladder are partially intact and partially destroyed (9) which may be due to either adverse effects of PDT or to successfully treated tumour masses that previously infiltrated and destroyed the wall of the urinary bladder. (Bars indicate 0.25 mm)

© 1999 Cancer Research Campaign at the position '0 J cm$^{-1}$' (controls). PDT using the lowest (0.3 mg kg$^{-1}$) or the middle (3 mg kg$^{-1}$) drug doses did not induce more damage to normal organs than found in control animals. In contrast, the highest photosensitizer dose (30 mg kg$^{-1}$) induced more damage to normal tissues compared to controls when optical doses of 700 J cm$^{-1}$ or more were used. These data suggest that a therapeutic window for PEG-m-THPC mediated PDT does exist where tumour necrosis occurs (Figure 1) and normal tissues are spared (Figure 2).

Figure 3 shows two representative H&E stained micrographs (10× magnification) of cross-sections through the lower abdomen of a rat sacrificed 3 days following PDT. The optical dose was 700 J cm$^{-1}$ fibre-length and the drug dose was 30 mg kg$^{-1}$ PEG-m-THPC. Both micrographs show completely necrotic tumour masses (1) encompassing normal pelvic organs such as the colon (2), the ureter (3) and the urinary bladder (4). Necrosis is surrounded by a strong inflammatory reaction (5). Although the light-diffuser was in the immediate vicinity of the pelvic organs, they show intact epithelial layers (6) with oedema of the underlying connective tissue (7). Musculature (8) of colon and ureter is histologically intact. The muscle layers of the urinary bladder (9) are partially destroyed. This may be due to an adverse effect of PDT on healthy tissue or due to successfully treated tumour masses that previously infiltrated and destroyed the wall of the urinary bladder.

Interestingly, hollow organs such as ureters, major blood vessels and colon remained patent even when severely damaged. No sequel of perforation such as uroperitoneum, peritonitis or haematoperitoneum were seen at necropsy. Increasing the optical dose from 700 J cm$^{-1}$ to 900 J cm$^{-1}$ did not significantly increase the volume of necrosis, but recovery of rats sensitized with 30 mg kg$^{-1}$ PEG-m-THPC was substantially slower when treated with 900 J cm$^{-1}$ than with 700 J cm$^{-1}$. Two out of four rats treated with the highest optical and drug dose did not survive the procedure. At necropsy no major internal haemorrhage was found in these animals, suggesting that the pronounced oedema observed induced severe fluid and electrolyte shifts leading to death. Following PDT, many rats were limping with the right hind-limb. The degree of limping appeared to be proportional to the drug–light dose product, and symptoms disappeared within three days. Hence, we assume that the limping was induced by swelling of the muscle and the connective tissue rather than by neural damage or myolysis. Most rats experienced slight to moderate bleeding from the branch canal when the light diffuser was withdrawn after completed irradiation. Bleeding was fatal in four of 55 rats.

The cumulative survival of untreated and treated tumour-bearing rats is shown in Figure 4 as a function of time (days). Mean overall survival for untreated tumour-bearing rats was 25.0 ± 4.5 days, whereas mean overall survival of treated rats was 38.4 ± 3.8 days and 40.0 ± 3.6 days for tumour-bearing rats exposed to 3 or 9 mg kg$^{-1}$ PEG-m-THPC-mediated minimally-invasive PDT respectively. Prolongation of survival with PDT was significant ($P < 0.05$) for both treated groups compared to controls. However, PDT using 9 mg kg$^{-1}$ PEG-m-THPC did not significantly prolong survival compared to the group treated with 3 mg kg$^{-1}$. All rats treated with PDT survived for at least 2 weeks, suggesting a lack of toxicity. Neither limb gangrene nor permanent limb paralysis were observed. All rats exposed to PDT excreted regularly. Macro-haematuria or blood containing faeces were not found. These observations indicate that despite the high likelihood for exposure to laser-light, blood-vessels, nerves, colon, ureters and urinary bladder remained functional. The skin at the fibre-insertion-site and next to the anus (i.e. where the fibre emerged) was always directly exposed to laser light. Surprisingly, none of the animals showed necrosis in either area, suggesting a lack of skin photosensitization 8 days after drug administration. Delayed wound healing at the fibre-insertion-site was not observed.

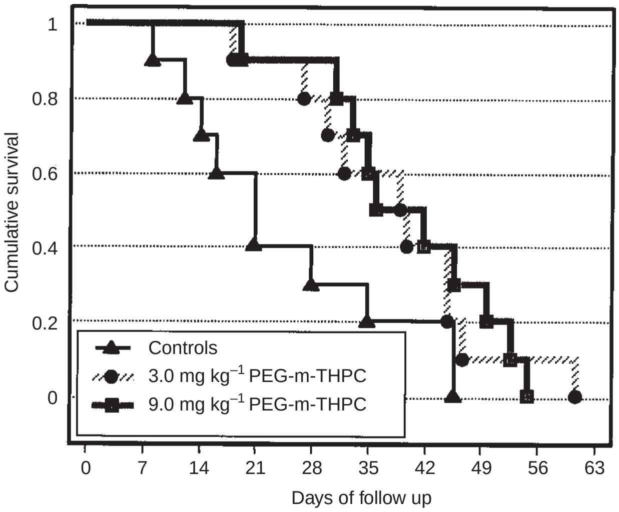

**Figure 4** The cumulative survival (Kaplan–Meier survival analysis) of untreated tumour-bearing rats and tumour-bearing rats treated with PDT is shown as a function of time (days). The follow-up of the rats started four weeks following tumour induction, when bulky tumours had developed. Thirty rats were randomly assigned (day 0) to either a control group ($n$ = 10), or groups injected with either 3 mg kg$^{-1}$ ($n$ = 10), or 9 mg kg$^{-1}$ PEG-m-THPC ($n$ = 10). An optical dose of 900 J cm$^{-1}$ of laser light in both PDT groups was applied blindly to the pelvis in a minimally-invasive procedure (day 8). Rats treated with PDT showed a significant prolongation of survival ($P < 0.05$ for both treated groups). PDT with 9 mg kg$^{-1}$ PEG-m-THPC showed a non-significant tendency to be more efficient than PDT with 3 mg kg$^{-1}$ PEG-m-THPC

### DISCUSSION

PDT has many theoretical advantages compared to conventional treatment modalities for cancer: PDT can be repeated without increased toxicity, it kills cancer cells by a distinct photochemical mechanism, and the risk of generating secondary cancer is small since the extent of DNA damage seems to be limited (Moan and Berg, 1992). Most important is the potential to destroy malignancies selectively if the photosensitizer is retained and/or accumulated preferentially in malignant tissue.

The poor prognosis of advanced ovarian cancer and recent developments in photomedicine have generated a considerable interest in PDT for this disease. Tochner et al (Tochner et al, 1985, 1986) have successfully treated small ovarian cancer deposits on the peritoneal surface with laser-light activated haematoporphyrin derivative (HpD) in mice. Similarly, intraperitoneal benzoporphyrin derivative mono-acid ring A (BPD-MA)-mediated PDT has been used to treat epithelial ovarian carcinomatosis in a mouse model, resulting in prolongation of survival (Molpus et al, 1996). Clinical phase I studies demonstrated promising results in patients treated with dihaematoporphyrin ethers (DHE) during open surgery for refractory or recurrent, disseminated intraperitoneal tumours (Sindelar et al, 1991; DeLaney et al, 1993). A recent study presented encouraging preliminary data on patients with peritoneal carcinomatosis due to recurrent ovarian cancer treated with laparoscopically guided PDT using m-THPC (Wierrani et al, 1997). All of these studies focused on eliminating small volumes of diffuse residual disease. In contrast, the present study assessed the effect of PDT on non-resectable large volume ovarian cancer bulks in the pelvis. Earlier studies reported significant morbidity after intra-abdominal PDT, including perforation in the gastrointestinal tract and necrotizing pancreatitis. These adverse effects were attributed to inadequate photosensitizer selectivity and/or light overdose (Lilge et al, 1998). Unusual high tumour selectivity of the photosensitizer PEG-m-THPC has been shown in a previous fluorescence microscopy study in the same tumour model (manuscript in preparation) eight days following systemic application. In the present study we showed selective tumour destruction using moderate drug concentrations which resulted in prolongation of survival, suggesting selective tumour targeting of PEG-m-THPC. Only the extremely high drug concentration of 30 mg $kg^{-1}$ activated by an optical dose of 700 J $cm^{-1}$ or more induced significant damage to normal organs, as demonstrated in the histological study (Figure 2). Hence, PDT with this photosensitizer seems to have a broad therapeutical window. To induce tumour necrosis a minimal drug dose of 3 mg $kg^{-1}$ body weight and a minimal optical dose of 300 J $cm^{-1}$ diffusing fibre was required.

Interstitial PDT was performed by placing the light diffusers blindly into the pelvis. If a non-selective photosensitization would have occurred, severe damage to normal tissues would be expected, with poor survival. It is important to note that normal organs in treated animals were exposed to significant optical doses of laser-light as indicated by necrotic tumour masses encompassing these organs. In fact, using moderate drug doses damage to normal tissues in treated rats was equal to controls. Further evidence for tumour selectivity of PDT is provided by the survival study (Figure 4). All treated animals survived for at least 2 weeks, and excreted regularly during that time, indicating functional integrity of the pelvic organs. Minimally-invasive PEG-m-THPC mediated PDT of rats bearing pelvic cancer resulted in significant prolongation of survival compared to tumour-bearing controls. Our findings are in good agreement with those reported by other groups. Ris et al (1998) demonstrated that PEG-m-THPC mediated PDT did not alter normal minipig bronchi, unlike the equimolar dose of the parent compound which induced ulceration and necrosis of bronchial mucosa. Westermann et al (1998) found a tumour to skeletal-muscle ratio of almost 20 for radioactive-labelled PEG-m-THPC whereas the radioactive-labelled parent compound reached only a ratio of 6. All of these researchers investigated drug-light-intervals up to 4 days. Our data strongly suggest that prolongation of the drug–light interval to 8 days may further enhance tumour selectivity while the strong PDT effect persists. This observation can be explained in part by the twofold longer half-life of PEG-m-THPC in the blood circulation compared to free m-THPC as described by Westermann et al (1998). Similarly, pegylation of liposomes seems to extend their longevity in circulation whereas their uptake by tumours is still possible due to increased microvascular permeability (Gabizon and Papahadjopoulos, 1988). For liposomes carrying drugs such as doxorubicin, it has been shown that pegylation increases selective accumulation in tumours and enhances anti-tumour activity (Gabizon et al, 1994).

Vascular shutdown is frequently seen with various photosensitizers. Neither our histology study nor the follow-up of treated rats in the survival study gave evidence for vascular shutdown. We consider this as a potentially important observation. Intact blood and oxygen supply enhances efficacy of subsequent chemotherapy, radiotherapy, or repeated PDT (Wouters and Brown, 1997). PDT using PEG-m-THPC may therefore allow combination with established treatment modalities.

For translation of this therapeutic concept into clinical use several adaptations are required. To debulk large tumour volumes, several fibres with diffusing tips of varying lengths could be inserted. The placement of laser fibres could be performed under guidance of an imaging system such as ultrasound, computerized tomography scan, or magnetic resonance imaging. Variation of irradiation parameters could further optimize dosimetry in complex tumour geometry. We did not observe skin lesions at the fibre insertion site following PDT, suggesting low skin photosensitization. However, there is currently no data on skin photosensitization of PEG-m-THPC available. Thus, sensitized patients need to be protected from intense light.

We conclude that minimally-invasive PEG-m-THPC-mediated PDT has a favourable therapeutic window and can reduce pelvic cancer bulks effectively and selectively. This approach deserves further investigation as treatment of non-resectable cancer. The method may be suitable for tumour debulking during open surgery or as a minimally-invasive procedure and may be combined with subsequent chemo- or radiotherapy.

### ACKNOWLEDGEMENTS

This work was made possible, in part, through access to the Laser Microbeam and Medical Program (LAMMP) and the Chao Cancer Centre Optical Biology Shared Resource at the University of California, Irvine. These facilities are supported by the National Institutes of Health under grants RR-01192 and CA-62203, respectively. Support was also provided by the Department of Energy (DOE #DE-FG03-91ER61227), and the NIH Institute of General Medical Sciences (GM-50958). R.H. wishes to acknowledge the Swiss National Research Foundation, the Huggenberger-Bischoff Stiftung, and the Theodor und Ida Herzog-Egli Stiftung. We wish to thank Scotia Pharmaceuticals, Ltd for their generous gift of PEG-m-THPC.

### REFERENCES

Barr H, Tralau CJ, Boulos PB, MacRobert AJ, Krasner N, Phillips D and Bown SG (1990) Selective necrosis in dimethylhydrazine-induced rat colon tumors using phthalocyanine photodynamic therapy. *Gastroenterology* **98**: 1532–1537

Ben-Hur E and Rosenthal I (1985) The phthalocyanines: a new class of mammalian cells photosensitizers with a potential for cancer phototherapy. *Int J Radiat Biol Relat Stud Phys Chem Med* **47**: 145–147

Bonnett R and Berenbaum M (1989) Porphyrins as photosensitizers. *Ciba Found Symp* **146**: 40–53

Bonnett R, White RD, Winfield UJ and Berenbaum MC (1989) Hydroporphyrins of the meso-tetra(hydroxyphenyl)porphyrin series as tumor photosensitizers. *Biochem J* **261**: 277–280

Boring CC, Squires TS, Tong T and Montgomery S (1994) Cancer statistics, 1994. *CA Cancer J Clin* **44**: 7–26

Brand E and Pearlman N (1990) Electrosurgical debulking of ovarian cancer: a new technique using the argon beam coagulator. *Gynecol Oncol* **39**: 115–118

Brand E, Wade ME and Lagasse LD (1988) Resection of fixed pelvic tumors using the Nd:YAG laser. *J Surg Oncol* **37**: 246–251

Chatlani PT, Nuutinen PJ, Toda N, Barr H, MacRobert AJ, Bedwell J and Bown SG (1992) Selective necrosis in hamster pancreatic tumours using photodynamic therapy with phthalocyanine photosensitization. *Br J Surg* **79**: 786–790

 © 1999 Cancer Research Campaign

Curtin JP, Malik R, Venkatraman ES, Barakat RR and Hoskins WJ (1997) Stage IV ovarian cancer: impact of surgical debulking [see comments]. *Gynecol Oncol* **64**: 9–12

Delaney TF and Glatstein E (1988) Photodynamic therapy of cancer. *Compr Ther* **14**: 43–55

DeLaney TF, Sindelar WF, Tochner Z, Smith PD, Friauf WS, Thomas G, Dachowski L, Cole JW, Steinberg SM and Glatstein E (1993) Phase I study of debulking surgery and photodynamic therapy for disseminated intraperitoneal tumors. *Int J Radiat Oncol Biol Phys* **25**: 445–457

DeLaney TF, Smith PD, Thomas GF, Tochner ZA, Sindelar WF, Pass HI, Harrington FS, Bonner RF and Mitchell JB (1991) A light-diffusing device for intraoperative photodynamic therapy in the peritoneal or pleural cavity. *J Clin Laser Med Surg* **9**: 361–366

Deppe G, Malviya VK, Boike G and Hampton A (1986). Surgical approach to diaphragmatic metastases from ovarian cancer. *Gynecol Oncol* **24**: 258–260

Deppe G, Malviya VK, Malone JJ and Christensen CW (1990) Debulking of pelvic and para-aortic lymph node metastases in ovarian cancer with the cavitron ultrasonic surgical aspirator. *Obstet Gynecol* **76**: 1140–1142

Gabizon A and Papahadjopoulos D (1988) Liposome formulations with prolonged circulation time in blood and enhanced uptake by tumors. *Proc Natl Acad Sci USA* **85**: 6949–6953

Gabizon A, Catane R, Uziely B, Kaufman B, Safra T, Cohen R, Martin F, Huang A and Barenholz Y (1994) Prolonged circulation time and enhanced accumulation in malignant exudates of doxorubicin encapsulated in polyethylene-glycol coated liposomes. *Cancer Res* **54**: 987–992

Gershenson DM (1994) Primary cytoreduction for advanced epithelial ovarian cancer. *Obstet Gynecol Clin North Am* **21**: 121–140

Goff BA, Hermanto U, Rumbaugh J, Blake J, Bamberg M and Hasan T (1994) Photoimmunotherapy and biodistribution with an OC125-chlorin immunoconjugate in an in vivo murine ovarian cancer model. *Br J Cancer* **70**: 474–480

Goff BA, Blake J, Bamberg MP and Hasan T (1996) Treatment of ovarian cancer with photodynamic therapy and immunoconjugates in a murine ovarian cancer model. *Br J Cancer* **74**: 1194–1198

Gomer CJ (1991) Preclinical examination of first and second generation photosensitizers used in photodynamic therapy. *Photochem Photobiol* **54**: 1093–1107

Gomer CJ and Dougherty TJ (1979) Determination of [$^{3}$H]- and [$^{14}$C]hematoporphyrin derivative distribution in malignant and normal tissue. *Cancer Res* **39**: 146–151

Grahn MF, McGuiness A, de Jode ML, Giger A, Dhiman AS, Cheung C-M, Pavitt S, Benzie R and Williams NS (1997) In vivo photodynamic activity of mTHPC poly(ethylene glycol) conjugates (SC102). *SPIE Proc* **3191**: 180–186

Kaplan E and Meier P (1958) Non-parametric estimation from incomplete observations. *Am Stat Assoc J* **53**: 457–480

Kimel S, Tromberg BJ, Roberts WG and Berns MW (1989) Singlet oxygen generation of porphyrins, chlorins, and phthalocyanines. *Photochem Photobiol* **50**: 175–183

Lilge L, Molpus K, Hasan T and Wilson BC (1998). Light dosimetry for intraperitoneal photodynamic therapy in a murine xenograft model of human epithelial ovarian carcinoma. *Photochem Photobiol* **68**: 281–288

Lowdell CP, Ash DV, Driver I and Brown SB (1993) Interstitial photodynamic therapy. Clinical experience with diffusing fibres in the treatment of cutaneous and subcutaneous tumours. *Br J Cancer* **67**: 1398–1403

Moan J and Berg K (1992) Yearly review; photochemotherapy of cancer: experimental research. *Photochem Photobiol* **55**: 931–948

Molpus KL, Kato D, Hamblin MR, Lilge L, Bamberg M and Hasan T (1996) Intraperitoneal photodynamic therapy of human epithelial ovarian carcinomatosis in a xenograft murine model. *Cancer Res* **56**: 1075–1082

Munkarah AR, Hallum AVr, Morris M, Burke TW, Levenback C, Atkinson EN, Wharton JT and Gershenson DM (1997) Prognostic significance of residual disease in patients with stage IV epithelial ovarian cancer [see comments]. *Gynecol Oncol* **64**: 13–17

Parker SL, Tong T, Bolden S and Wingo PA (1996) Cancer statistics, 1996 [see comments]. *CA Cancer J Clin* **46**: 5–27

Pass HI (1993) Photodynamic therapy in oncology: mechanisms and clinical use. *J Natl Cancer Inst* **85**: 443–456

Peng Q, Moan J, Ma LW and Nesland JM (1995) Uptake, localization, and photodynamic effect of meso-tetra(hydroxyphenyl)porphine and its corresponding chlorin in normal and tumor tissues of mice bearing mammary carcinoma. *Cancer Res* **55**: 2620–2626

Pettersson F (1995) *Annual Report in the Results of Treatment in Gynecological Cancer*, Vol. 22. Radiumhemmet: Stockholm.

Ris HB, Altermatt HJ, Inderbitzi R, Hess R, Nachbur B, Stewart JC, Wang Q, Lim CK, Bonnett R, Berenbaum MC and et al. (1991) Photodynamic therapy with chlorins for diffuse malignant mesothelioma: initial clinical results. *Br J Cancer* **64**: 1116–1120

Ris HB, Altermatt HJ, Nachbur B, Stewart JC, Wang Q, Lim CK, Bonnett R and Althaus U (1993*a*) Effect of drug–light interval on photodynamic therapy with metatetrahydroxyphenylchlorin in malignant mesothelioma. *Int J Cancer* **53**: 141–146

Ris HB, Altermatt HJ, Stewart CM, Schaffner T, Wang Q, Lim CK, Bonnett R and Althaus U (1993*b*) Photodynamic therapy with m-tetrahydroxyphenylchlorin in vivo: optimization of the therapeutic index. *Int J Cancer* **55**: 245–249

Ris HB, Im Hof V, Stewart CM, Mettler D and Altermatt HJ (1998) Endobronchial photodynamic therapy: comparison of mTHPC and polyethylene glycol-derived mTHPC on human tumor xenografts and tumor-free bronchi of minipigs. *Lasers Surg Med* **23**: 25–32

Rose GS, Tocco LM, Granger GA, DiSaia PJ, Hamilton TC, Santin AD and Hiserodt JC (1996) Development and characterization of a clinically useful animal model of epithelial ovarian cancer in the Fischer 344 rat. *Am J Obstet Gynecol* **175**: 593–599

Rosenthal I (1991) Phthalocyanines as photodynamic sensitizers. *Photochem Photobiol* **53**: 859–870

Schwartz PE (1997) Cytoreductive surgery for the management of stage IV ovarian cancer [editorial; comment]. *Gynecol Oncol* **64**: 1–3

Sindelar WF, DeLaney TF, Tochner Z, Thomas GF, Dachoswki LJ, Smith PD, Friauf WS, Cole JW and Glatstein E (1991) Technique of photodynamic therapy for disseminated intraperitoneal malignant neoplasms. Phase I study. *Arch Surg* **126**: 318–324

Testa JR, Getts LA, Salazar H, Liu Z, Handel LM, Godwin AK and Hamilton TC (1994) Spontaneous transformation of rat ovarian surface epithelial cells results in well to poorly differentiated tumors with a parallel range of cytogenetic complexity. *Cancer Res* **54**: 2778–2784

Tochner Z, Mitchell JB, Harrington FS, Smith P, Russo DT and Russo A (1985) Treatment of murine intraperitoneal ovarian ascitic tumor with hematoporphyrin derivative and laser light. *Cancer Res* **45**: 2983–2987

Tochner Z, Mitchell JB, Smith P, Harrington F, Glatstein E, Russo D and Russo A (1986) Photodynamic therapy of ascites tumours within the peritoneal cavity. *Br J Cancer* **53**: 733–736

Tochner Z, Mitchell JB, Hoekstra HJ, Smith P, DeLuca AM, Barnes M, Harrington F, Manyak M, Russo D, Russo A and et al. (1991) Photodynamic therapy of the canine peritoneum: normal tissue response to intraperitoneal and intravenous photofrin followed by 630 nm light. *Lasers Surg Med* **11**: 158–164

Veenhuizen RB, Ruevekamp-Helmers MC, Helmerhorst TJ, Kenemans P, Mooi WJ, Marijnissen JP and Stewart FA (1994) Intraperitoneal photodynamic therapy in the rat: comparison of toxicity profiles for photofrin and MTHPC. *Int J Cancer* **59**: 830–836

Weishaupt KR, Gomer CJ and Dougherty TJ (1976) Identification of singlet oxygen as the cytotoxic agent in photoinactivation of a murine tumor. *Cancer Res* **36**: 2326–2329

Westerman P, Glanzmann T, Andrejevic S, Braichotte DR, Forrer M, Wagnieres GA, Monnier P, van den Bergh H, Mach JP and Folli S (1998) Long circulating half-life and high tumor selectivity of the photosensitizer meta-tetrahydroxyphenylchlorin conjugated to polyethylene glycol in nude mice grafted with a human colon carcinoma. *Int J Cancer* **76**: 842–850

Wierrani F, Fiedler D, Grin W, Henry M, Dienes E, Gharehbaghi K, Krammer B and Grunberger W (1997) Clinical effect of meso-tetrahydroxyphenylchlorine based photodynamic therapy in recurrent carcinoma of the ovary: preliminary results. *Br J Obstet Gynaecol* **104**: 376–378

Wouters BG and Brown JM (1997) Cells at intermediate oxygen levels can be more important than the ‘hypoxic fraction’ in determining tumor response to fractionated radiotherapy. *Radiat Res* **147**: 541–550

© 1999 Cancer Research Campaign 
